# Wellcome’s 2019 funding for PhD programme training: An experiment in enhancing research culture

**DOI:** 10.12688/wellcomeopenres.21052.1

**Published:** 2024-04-24

**Authors:** Annamaria Carusi

**Affiliations:** 1 Interchange Research, London, E5, UK

**Keywords:** research culture, research training, funding policy

## Abstract

In September 2019, the results of Wellcome’s call for proposals for PhD training programmes were announced. This funding call was notable for making contributions to positive research culture as important a selection criterion as scientific excellence in PhD training. Aware that it’s intervention on enhancing research culture was experimental, Wellcome also commissioned work to reflect on its processes. I undertook two studies of the early phases of the funding call, of the Wellcome’s internal processes in shaping and implementing the call, and of the experiences and reflections of all applicants to the call. This Open Letter is a summary of the cross cutting themes of these two studies: the difficulties of defining positive research culture and of balancing it with scientific excellence; the expectation that supervision of PhD students is a key way to effect research culture change; the need for coherence between funding calls; issues around evaluating the track records of programmes on research culture, which further feed into defining the criteria of evaluation and selection relating to research culture.

## Plain Language Summary

For some time, it has been widely acknowledged that the culture of scientific research is negative, some would even say ‘toxic’. There are many factors contributing to negative research culture, many of them systemic or structural, rather than having to do with individual scientists. These factors can make it more difficult to behave in ways that get in the way of best practices in research (such as sharing data and collaborating) and easier to behave in ways that are detrimental to research and to the well being of the research community, such as being aggressively competitive. There are a great many issues that fall under research culture, from addressing bullying and harassment, all the way to open science and scientific integrity. In September 2019, 23 Wellcome funded PhD programmes were launched. These programmes had been funded in a call that for the first time put positive research culture on a par with scientific excellence. This was a bold and experimental intervention in the research system. Aware of this fact, Wellcome also commissioned two studies into the shaping and implementation of the call, and into the way it had been received by applicants to the call. This Open Letter summarises the main themes that cut across the two studies: the difficulties of defining positive research culture and of balancing it with scientific excellence; the expectation that supervision of PhD students is a key way to effect research culture change; the need for coherence between funding calls; issues around evaluating the track records of programmes on research culture, which further feed into defining the criteria of evaluation and selection relating to research culture.

## Disclaimer

The views expressed in this article are those of the author(s). Publication in Wellcome Open Research does not imply endorsement by Wellcome.

In September 2019, the results of Wellcome’s call for proposals for PhD training programmes were announced ^1^. This funding call was notable for making contributions to positive research culture as important a selection criterion as scientific excellence in PhD training. It was a bold move, which Wellcome was in a good position to make due to its history of innovation in PhD training (it was the first funder to have introduced graduate training in cohorts), and due to its unique position in the UK research funding landscape. Although it funds only a relatively small percentage of biomedical PhD studentships, it is an influential funder. We currently see the UKRI making similar moves in giving research culture a greater priority in the evaluation of research institutions, which has given rise to much discussion and controversy.

Aware that it’s intervention on enhancing research culture was experimental, Wellcome also commissioned work to reflect on its processes. I undertook two studies of the early phases of the funding call, of the Wellcome’s internal processes in shaping and implementing the call, and of the experiences and reflections of all applicants to the call. This Open Letter is a summary of the cross cutting themes of these two studies: the difficulties of defining positive research culture and of balancing it with scientific excellence; the expectation that supervision of PhD students is a key way to effect research culture change; the need for coherence between funding calls; issues around evaluating the track records of programmes on research culture, which further feed into defining the criteria of evaluation and selection relating to research culture.

Wellcome has a headstart on other funders in using a funding tool to intervene in research culture and attempt to change it. As policies to improve research culture are currently being shaped in the UK, much can be learned from its experience and example. My hope is that this Open Letter will bring these experiences to the fore, and contribute to the broader discussion of research culture, and how to intervene to bring about much needed improvements.

Part One ( Carusi, 2024a) of this study focused on the internal processes of the Wellcome Trust in shaping and implementing the 2019 PhD Programme call. Interviews were conducted with Wellcome Trust staff involved in different capacities with the decision making and roll out of the call; and a discussion focus group was held with the members of the selection committee after the final selection meeting.

Part Two of the study ( Carusi, 2024b) focused on the applicants who responded to the call, with the aim of understanding how they interpreted the call, and their experience of the submission and selection process. This was conducted via a survey, and interviews with some respondents to the survey ^2^.

Each of the two reports is preceded by a discussion of the main themes that emerged from the respective studies, which I will not repeat here. In the points below I bring out the main points of convergence between those who shaped and implemented the call, and those who applied to it, whether successful or not.

- While generally study participants in both parts of this study identified with the value of positive research culture, what this actually meant was not fixed and stable, and was subject to disagreement and controversy. While participants often responded with a list of factors that come under the remit of research culture, when each of the factors was discussed, it was clear that there were differences in what they were considered to apply to in practice: for example, whether these are factors that demand particular characteristics from programme directors and supervisors (such as being more nurturing, or being versed in the different kinds of mental health issues students might suffer from), or whether they are factors that institutions should take care of through wellbeing or careers services in place for students? Are they an inherent aspect of science and how students are trained, or are they marginal to science? How broadly should diversity and inclusion be interpreted? When are some forms of behaviour to be considered bullying? And so on, each of the listed criteria for a positive research culture could in fact take many different forms, and could be considered the responsibility of different actors.
- The balance between scientific excellence and positive research culture in evaluating and selecting the applications was particularly controversial, both within the Wellcome Trust, and among the applicants. This issue was the most divisive of the whole process. While everyone could embrace a positive research culture as a laudable aim, there were sharp divisions when it came to placing it on a par with scientific excellence. While some felt that this would allow them to seize an opportunity to shape doctoral training according to values that they aspired to, for others this risked devaluing science.
- Supervision is the primary relationship around which research culture is enacted; it is a two-way conduit for expectations of behaviour between academics and students. The relationship is shaped by the values of all actors involved in it, and of the pressures on all of them too. For example, training students for careers in science is a strong motivator for academic supervisors through which they express the high value that they place on their discipline or on science in general, and while the reality of scarcity of academic careers might force them to consider other destinations for students, this often does not speak directly to their value system. Or, on a contrasting note, student-centredness is not neutral for supervisors as it may detract from their ability to deliver on their own funded research. The study of applicants’ responses to the call showed the extent to which research culture for training is particularly embedded in this relationship, and needs to take into account supervisors’ needs and values, and the context of research for supervisors.
- At the Wellcome Trust there is a great deal of reflection on research culture, as seen in this call, but also more generally in the organisation through, for example, the broader studies being conducted on this aspect of research life. However, this can still be quite far from the experience of academics, who are attempting to respond to calls to improve research culture while themselves being in an institutional context that makes this very difficult. In some cases, this is in part due to the way in which funding by the Wellcome Trust of individuals and centres shapes their own local institutional context and the support available to them. Individuals could find themselves at cross purposes between funding strands. The need for coherence between the different strands of funding was a recurring point made by applicants.
- The call for greater clarity, and for defined criteria was also heard in both contexts, internal to the Wellcome Trust and among applicants. Along with this, for the applicants, went calls for greater transparency regarding how decisions were made, in particular because of the immense amount of time and effort that went into the applications, and because of the high failure rate.
- This is tied in with the definitions of positive research culture, with many applicants saying that they would have liked to have seen examples of best practice. However, this was not available to this call, since the approach taken was not to be overly prescriptive and to allow applicants the freedom to innovate. The call for clarity regarding what would count as a positive research culture, or as steps to improve research culture resonated across Wellcome staff and applicants. Two inter-related questions that emerged in both studies are: what constitutes best practices in a systems approach, that is, when these are not pre-defined? And how can clarity be achieved when best practices are considered emergent rather than pre-defined?
- This is also connected to the selection process, with the panel and applicants both noting that it was not clear why it should be a two stage application, given that there was not enough difference between the applications at the two stages, and feedback after preliminary stage did not sufficiently highlight the points that would count against applications at final stage, or even sometimes contradicted feedback after the final selection. Issues around selection for funding continue to exercise the funding and research communities; in this regard it is interesting to note that Wellcome’s most recent funding of research culture projects used partial randomisation in the selection process ( Lewis-Wilson *et al.* (2023). This was a strategic choice, as Wellcome purposely avoids imposing a definition of best practices, choosing instead to provide scope for communities to define these for themselves.

In ongoing initiatives, Wellcome has also commissioned the Emerging Research Cultures project, led by me, to form a community of practice consisting of all staff and students of the 23 funded PhD programmes. This is to provide an opportunity to share experiences and learnings from the programmes. One thing was abundantly clear: that there was not a clear definition or understanding of research culture provided at the outset. Such an *a priori* definition would most probably have been counterproductive, as it would have excluded the bottom-up emergence of understandings of what falls under the rubric of research culture, how different aspects of it are prioritised in different settings, how different programmes have acted to try to enhance the aspects that seemed most important to them, or most amenable to being changed. There are now several reports describing the processes and practices of the different programmes, and the students’ and staff’s thoughts on these, on the Emerging Research Cultures website. A second point of clarity is that research culture change takes time. The experiment is still evolving, even as there are lessons to be learned from it.

## Ethics and consent

Ethical approval and consent were not required.

## Data Availability

No data are associated with this article.
