# Supplementary material for: Wellcome’s 2019 funding for PhD programme training: An experiment in enhancing research culture
Source: Wellcome Open Res. 2024 Apr 24;9:224. [Version 1] doi: 10.12688/wellcomeopenres.21052.1 (PMC11179046; doi:10.12688/wellcomeopenres.21052.1)
Supplement: Peer review history

# Reviewer report: Reviewer response for version 1

**DOI:** 10.21956/wellcomeopenres.23288.r84285

**Published:** 2024-06-14

**Authors:** Karri A. Holley

**Affiliations:** 1 The University of Alabama, Tuscaloosa, Alabama, USA

The open letter reports on studies of Wellcome’s call for proposals with an emphasis on a positive research culture. Such a call is aligned with those of other agencies and foundations globally that increasingly recognize the roles of the supervisor, the faculty, and the institution in the doctoral student experience (e.g., the US National Science Foundation’s recent shift to require principal investigators to include graduate students in mentoring plans for all submitted proposals). A strength of this open letter is its accessibility to a range of audiences, especially with clear methodological explanations and reflections from those who constructed the call and those that responded to it.

 The findings reported here are of value to those concerned about how to best support graduate students in the research enterprise. The letter raises questions that may have divergent answers, depending on the audience, and the answers have clear implications for scientific behavior and practice. How such cultures should be assessed and in what ways they might influence the definition of scientific excellence is a problematic point for all entities in the process, including the external funding agency, which the letter emphasizes. More attention might have given here to what the divergence means, especially for faculty as mentors and research leaders. Depending on a range of individual factors (employment status, academic discipline, etc.), faculty might have differing approaches towards defining and facilitating a positive research culture. Managing and responding to these differing approaches then is an obligation placed on academic institutions and funding agencies.

 Of interest for Wellcome, academic institutions, faculty, and students is the challenge of defining a positive research culture. The open letter addresses this challenge at numerous points, providing guideposts for future exploration. How might we reconcile the desire for Wellcome not to be overly prescriptive in its approach to a positive research culture, while responding to applicants who seek greater clarity and defined criteria? What does a positive research culture look like across institutions, disciplines, research settings, and global contexts? How might such a definition respond to variations? Can the a priori definition exist at the same time with bottom-up, emergent concepts? The letter and its related report provide an important foundation for advancing these questions.

Does the article adequately reference differing views and opinions?

Yes

Are all factual statements correct, and are statements and arguments made adequately supported by citations?

Yes

Is the Open Letter written in accessible language?

Yes

Where applicable, are recommendations and next steps explained clearly for others to follow?

Yes

Is the rationale for the Open Letter provided in sufficient detail?

Yes

Reviewer Expertise:

Graduate and doctoral education. curriculum, organizational change in higher education

I confirm that I have read this submission and believe that I have an appropriate level of expertise to confirm that it is of an acceptable scientific standard.

# Reviewer report: Reviewer response for version 1

**DOI:** 10.21956/wellcomeopenres.23288.r84288

**Published:** 2024-06-08

**Authors:** Rosemary Deem

**Affiliations:** 1 Royal Holloway University of London, Egham, England, UK

This Open Letter reports on two concise and well-conducted pieces of research relating to the development of and response to an experimental call in 2019 from the Wellcome Trust for funding of PhD training programmes in biomedical science, in which both research cultures and scientific excellence were selection criteria.  The nature of research cultures experienced by doctoral researchers that exist in UK universities is a topical theme to which Wellcome has particularly drawn attention (Moran & Wild, 2019 ). The two studies here, one focused on internal processes of the Wellcome Trust in preparing and evaluating the 2019 call and the other on the experiences of those applying for the training programmes, present a balanced and clearly explained account of issues raised by different participants.  Aspects of the 2019 call such as applicants’ responses to the two stage application process and transparency and consistency in giving feedback are also explored.  The Letter further discusses varied views (from both Wellcome and applicants) on concerns like the difficulty of defining what a positive research culture looks like and whether trying to both put in place a positive culture whilst also ensuring scientific excellence can coexist or are in conflict.  Some important points are made about the role and responsibility of doctoral supervisors for encouraging positive research cultures ( including considering who else in universities might also be responsible, for example in connection to mental health matters or careers advice beyond academe or trying to be student centred whilst still maintaining supervisors’ funded research projects). One aspect that is not explored in the Letter is the extent to which doctoral researchers are often supervised by more than one person, a trend evident in both the UK and beyond (Gower & Clegg, 2021; Pyhältö et al., 2023), leading to the possibility that members of the same supervisory team (some of whom may not be academics) may themselves have different views on research cultures, which may be confusing for doctoral researchers.   The findings about the experimental 2019 call and the two studies are certainly very valuable beyond the Wellcome Trust and the applicants to the 2019 call, such as, for example, UKRI as a major funder of doctoral training collaborations in the UK.   However, it is also worth considering that some aspects of the findings here about academics’ views may not be fully replicated in other disciplinary fields such as social sciences or arts and humanities, especially in relation to what constitutes a positive research culture and how that links to or conflicts with academic excellence.  Overall though, this is an important piece of research in the field of doctoral education and training, with sufficiently detailed  rationale,  covering multiple points of view, making some reference to citations to back up statements,  using accessible language and providing details of a follow up Emerging Researcher Cultures project.

UKCGE | UK Research Supervision Survey 2021 Report

what-researchers-think-about-the-culture-they-work-in-qualitative-research.pdf (wellcome.org)

Does the article adequately reference differing views and opinions?

Yes

Are all factual statements correct, and are statements and arguments made adequately supported by citations?

Yes

Is the Open Letter written in accessible language?

Yes

Where applicable, are recommendations and next steps explained clearly for others to follow?

Yes

Is the rationale for the Open Letter provided in sufficient detail?

Yes

Reviewer Expertise:

Doctoral education.  Equality, Diversity and Inclusivity

I confirm that I have read this submission and believe that I have an appropriate level of expertise to confirm that it is of an acceptable scientific standard.

## References

[1] : The more the merrier? PhD supervisors’ perspectives in engaging in co-supervision. *Innovations in Education and Teaching International* .2023; 10.1080/14703297.2023.2258853 1-12 10.1080/14703297.2023.2258853
